# Supplementary material for: Exploring the impact of nature connectedness on well-being and mental distress among urban youth: Evidence from 25 most populated cities in India
Source: PLoS One. 2025 May 30;20(5):e0323712. doi: 10.1371/journal.pone.0323712 (PMC12124530; doi:10.1371/journal.pone.0323712)
Supplement: Attachment — Submitted filename: Recomendations.pdf [file pone.0323712.s001.pdf]

Recomendações para Melhoria do artigo.

1- Address the issue of sampling bias in the text, including a more in-depth discussion of the implications of this bias on the results. Sample imbalance can affect the result and it would be important to detail the implications in the study.

2- Can there be seasonal variations in the collection of results? Does the data collected allow this to be visualized? If so, can this be explored in the paper?

3- Although the study acknowledges some limitations, such as the cross-sectional nature of the research and the possibility of bias in the data, the discussion of these issues is relatively brief, it would be important to include a more critical and in-depth analysis of the study's limitations and their potential implications for the conclusions. It is important to present the limitations transparently and discuss how they may influence the interpretation of the results.

4- The study focuses mainly on the statistical analysis of the relationship between connection with nature and mental well-being, it would be important to explore the practical implications of the study for urban planning, public mental health policies and community interventions. It is important to discuss how the results can be used to promote the mental health and well-being of the urban population through the creation of and access to green and blue spaces. Presenting the practical implications of the study makes the research more relevant and useful.
