# Supplementary material for: Exploring the impact of nature connectedness on well-being and mental distress among urban youth: Evidence from 25 most populated cities in India
Source: PLoS One. 2025 May 30;20(5):e0323712. doi: 10.1371/journal.pone.0323712 (PMC12124530; doi:10.1371/journal.pone.0323712)
Supplement: Attachment — Submitted filename: Response to reviewers.pdf [file pone.0323712.s002.pdf]

We want to thank the reviewers for their valuable suggestions in improving the article. We have added our responses to each comment below. We have also made relevant changes in the manuscript.

Reviewer 1

I reviewed the article titled "Exploring the impact of nature connectedness on well-being among urban youth from 25 most populated cities in India". It contributes significantly to the scientific literature in several ways:

1. This study addresses the lack of research on the relationship between nature connectedness and well-being among urban youth in India, a region where such studies are scarce. By focusing on a large and diverse sample from 25 major cities, the research provides a comprehensive understanding of how nature connectedness influences the mental health of urban youth in a rapidly urbanizing country.
2. The study employs robust statistical methods, including multivariate and logistic regression analyses, to explore the impact of residential exposure to green and blue spaces, frequency of recreational visits, and time spent indoors on well-being. This multifaceted approach offers nuanced insights into the various factors that contribute to mental health outcomes.
3. The findings have significant implications for urban planning and public health policies. By demonstrating the positive effects of proximity to green and blue spaces and frequent interaction with nature on mental well-being, the study advocates for the development of more parks and green spaces in urban areas. This evidence can be used to inform policies aimed at improving the mental health of urban populations, particularly the youth.
4. While the study is region-specific, its findings contribute to the broader global literature on the benefits of nature connectedness. By providing empirical evidence from a developing country context, the research adds a valuable perspective to the existing knowledge, which is predominantly based on studies from Western countries.
5. The study's results can be used by educators, mental health professionals, and urban planners to design interventions that promote nature connectedness as a means of enhancing mental well-being among youth. This practical application further underscores the importance of the research.

Based on the review of the study, below are the overall weaknesses identified which need the author(s) attention:

1. The study is based on responses from 25 cities, but the number of responses per city is uneven, ranging from 6 in Rajkot to 415 in Delhi. This uneven distribution could lead to sampling bias and may not accurately represent the youth populations in all cities.

   Thank you for your observation. We acknowledge that the number of responses collected across the 25 cities in our study is uneven, ranging from 6 in Rajkot to 415 in Delhi. This variation in response rates is influenced by factors such as the differing population sizes of the cities, the effectiveness of survey outreach, and logistical challenges in certain regions. For example, smaller cities like Rajkot, Srinagar, and Vadodara experienced more significant difficulties in reaching a larger sample size due to limited resources and outreach capabilities.

   While we recognize the potential for sampling bias, we believe this imbalance does not substantially affect the overall findings at the combined level. The primary focus of our analysis is on broader trends and patterns across the entire dataset, and city-wise analyses are considered secondary in this regard. Nonetheless, we have included this as a limitation in the study and recognize the need for more balanced sampling in future research. We are committed to addressing this challenge by employing better strategies in subsequent studies to ensure better representation across all cities.

2. The study uses a cross-sectional design, which limits the ability to draw causal inferences. It only captures a snapshot in time rather than observing changes over time, which could provide more robust evidence for the relationships explored.

Thank you for this suggestion. We acknowledge that we have not included a longitudinal research design with time series analysis in this work. However, we plan to explore this in future research, where we would consider employing models such as SARIMA or other appropriate techniques to capture temporal variations and provide a more comprehensive understanding of the relationships explored. We have noted this limitation in the relevant section of the manuscript.

3. The data relies entirely on self-reported measures, which can be prone to biases such as social desirability bias and recall bias, potentially leading to inaccurate reporting of nature connectedness and well-being.

   Thank you for highlighting the potential biases associated with self-reported measures. We acknowledge that social desirability bias and recall bias can indeed influence participants' responses, which may affect the accuracy of the reported levels of nature connectedness and well-being.

   To address these concerns, we took several steps to mitigate such risks. First, we ensured the anonymity of respondents to reduce the likelihood of socially desirable responses. Additionally, we employed well-established and validated scales for measuring nature connectedness and well-being, which are specifically designed to minimize bias and enhance the reliability of the data. Despite these precautions, we recognize that some level of bias may still be present and have noted this as a limitation in our study.

4. Since the data collection was conducted through an online survey, there is no way to validate the accuracy of the responses, leading to potential inaccuracies in the data.

   Thank you for raising the concern regarding the accuracy of responses in online surveys. We acknowledge that the lack of direct validation of responses can introduce potential inaccuracies in the data. To mitigate this issue, we implemented several measures. The survey was designed to be user-friendly and concise, minimizing the risk of response fatigue and careless reporting. Additionally, participants were recruited through verified channels, targeting individuals who were more likely to provide thoughtful and honest responses. While these steps help reduce potential inaccuracies, we recognize the inherent limitations of online survey data collection and have addressed this in the study's limitations section.

5. Some survey questions are described as ambiguous, which could lead to misinterpretation by participants and affect the validity of the results.

   Thank you for pointing out the potential for ambiguity in some of the survey questions. We appreciate the concern, and we believe we have made every effort to minimize ambiguity in the design of the survey. Several steps were taken to ensure clarity: First, many of the survey items were adapted from validated and widely used instruments that have been tested for both clarity and reliability in prior research. Additionally, a pilot survey was conducted with a small subset of the target population to identify and address any unclear or ambiguous questions before the main data collection. To further enhance clarity, we provided explanations or examples wherever necessary to help participants better understand potentially ambiguous terms.

6. The study focuses only on youth aged 18-25 years. This narrow age range limits the generalizability of the findings to other age groups, such as younger adolescents or older adults.

   Thank you for your comment regarding the age range in our study. We would like to clarify that this research specifically focuses on urban youth, as indicated in the title, and the age range of 18-25 years was chosen to align with this focus. While the findings of this study are limited to this demographic, future research could certainly explore the extension of the study to include other age groups, such as younger adolescents or older adults, to enhance the generalizability of the findings.

7. The study is limited to urban areas in India, and the findings may not be generalizable to rural areas or other countries with different cultural and environmental contexts.

   Thank you for your comment. As mentioned earlier, this study specifically focuses on urban youth, as indicated in the title, and therefore the data collection was limited to the 25 largest and most populated cities in India. The aim was to explore the well-being and nature connectedness of youth in urban environments, where cultural, environmental, and socio-economic factors may differ significantly from

rural areas or other countries. While the findings of this study are not directly generalizable to rural populations or other cultural contexts, future research could extend this work to include rural youth or populations in different countries.

8. The study does not account for seasonal variations, which could affect outdoor activities and nature connectedness. For instance, participants might engage more with nature during certain seasons, influencing their well-being.

   Thank you for highlighting the potential impact of seasonal variations on outdoor activities and nature connectedness. We acknowledge that this is a limitation of our study, as data collection was conducted during a specific period and did not account for seasonal differences. Due to logistical constraints and the time limitations of the study, it was not feasible to capture responses across multiple seasons. While this may introduce some bias, it is important to emphasize that the focus of this study was on broader trends rather than season-specific behaviors. In future research, we plan to incorporate longitudinal data collection across different seasons to more accurately capture seasonal variations and their potential impact on nature connectedness and well-being.

9. The study finds that proximity to blue spaces has an unexpected negative impact on well-being, but this result is not deeply explored. The lack of blue spaces in the sample could have skewed the data, but this issue is not adequately addressed.

   Thank you for your observation. We acknowledge that the finding regarding the unexpected negative impact of proximity to blue spaces on well-being warrants further exploration. In response, we have provided additional context and explanations in the discussion section to better address this result.

   Firstly, the number of individuals with residential proximity to blue spaces in urban areas is significantly lower compared to green spaces. The scarcity of blue spaces in many cities, particularly those that are not coastal or lack substantial water bodies such as lakes, ponds, or streams, results in limited exposure for the study participants. As a result, the data corresponding to blue space exposure may be skewed, with a relatively small sample size of individuals benefiting from access to blue spaces.

   Moreover, in urban settings, the quality of blue spaces may vary greatly, and proximity alone may not necessarily lead to positive mental health outcomes. For instance, if the blue space is poorly maintained, polluted, or inaccessible for recreational use, it may not have the intended restorative effects on well-being. Additionally, the perception of blue space as a calming and restorative environment may depend on its type and the surrounding urban infrastructure. This contrasts with green spaces, which, on average, tend to be more readily available, accessible, and perceived as beneficial for mental health. Future studies could explore the quality and types of blue spaces, including water bodies' accessibility, cleanliness, and surrounding areas, to better understand how blue spaces influence mental health and distress in urban youth.

   Although blue spaces may generally be considered beneficial for well-being, their limited availability and varied quality in urban environments may explain the unexpected findings in this study. Further research could focus on cities with greater access to blue spaces to offer a more comprehensive understanding of their impact on mental health.

10. While the study includes various covariates, it may not account for all potential confounding factors, such as socio-economic status, existing mental health conditions, or environmental factors unrelated to nature that could influence well-being.

    Thank you for your thoughtful comment. We acknowledge that, while our study includes a range of covariates, it may not account for all potential confounding factors that could influence well-being. In our analysis, we included key covariates such as age, gender, education level, and geographical location, which are known to affect both nature connectedness and well-being based on existing literature. However, we recognize that other factors, such as socio-economic status, pre-existing mental health conditions, and environmental influences unrelated to nature, could also play a significant role in shaping well-being. Due to data availability and scope limitations, these additional factors were not included in the current study. We acknowledge this as a limitation and suggest that future research could expand the range of covariates to better capture the full complexity of influences on well-being.

11. Although some limitations are acknowledged, the discussion might not be comprehensive enough, leaving out other potential weaknesses that could impact the study's validity.

    Thank you for your feedback. In response, we have thoroughly revised the discussion section to provide a more comprehensive analysis of the study's limitations. We have now included a dedicated subsection that outlines these limitations in detail. Additionally, we have highlighted key recommendations for future research to address these issues. We believe these revisions provide a clearer understanding of the study's constraints and offer direction for future work in this area.

12. The study relies heavily on quantitative data, with little to no qualitative insights that could provide a deeper understanding of the subjective experiences of nature connectedness and its impact on well-being.

    Thank you for your thoughtful comment. The primary aim of this study was to identify broad trends and associations across a large and diverse urban youth population in India, which is why we focused on quantitative data. This approach allowed us to systematically analyze patterns and draw statistically robust conclusions. However, we recognize that qualitative insights could offer a deeper understanding of the subjective experiences of nature connectedness and its impact on well-being.

    We appreciate your suggestion and agree that incorporating qualitative methods, such as in-depth interviews, focus groups, or open-ended survey questions, would be valuable in future research. We have acknowledged this as a limitation in the study and highlighted the potential for integrating qualitative approaches in future work to enrich the findings.

13. With 2,283 participants responding to an online survey, there is a risk of survey fatigue, where participants may not fully engage with the later questions, leading to incomplete or less thoughtful responses.

    Thank you for raising the concern about survey fatigue, which is indeed a potential risk in studies involving lengthy online surveys. To mitigate this, we took several measures: the survey was designed to be concise, focusing on key variables while minimizing the number of questions. We structured the survey in a logical and engaging manner to maintain participant interest throughout. Additionally, a pilot survey was conducted to ensure that the length and complexity were manageable for participants. These steps were aimed at reducing the likelihood of survey fatigue and ensuring thoughtful and complete responses.

14. The study may focus heavily on statistically significant results without thoroughly considering the practical significance or the effect sizes, which might be small even when statistically significant.

    Thank you for your observation. We agree that focusing solely on statistical significance can sometimes overlook the practical implications of the findings. To address this, we have made an effort to interpret the significant coefficients in the regression model and highlight their real-world relevance. For example, as shown in Table 2, we observe that access to a green space within 1 km, compared to no access, is associated with a 2.18 increase in the WHO score. While the statistical significance of this result is important, we also aim to provide context regarding its practical significance. We recognize the value of considering effect sizes and their implications for real-world applications and will continue to focus on both statistical and practical significance in future research.

In addition to the weaknesses mentioned above, below are suggested area of improvement divided section by section:

1. Title : Consider rephrasing the title to include the terms "urban mental health" or "youth well-being" to better capture the essence of the study.

   Based on your recommendation, we have revised the title to: Exploring the impact of nature connectedness on urban mental health and well-being among youth from 25 most populated cities in India. This revised title better captures the essence of the study and aligns with its central themes.

2. Abstract: Add a brief mention of key limitations in the abstract to provide a more balanced overview of the study's contributions and its constraints.

Thank you for your suggestion. We have incorporated a brief mention of the key limitations in the abstract as follows: The study does, however, have limitations, including uneven sample distribution among cities, the absence of temporal analysis, and a restricted age range of 18-25 years. These limitations will be addressed in future research.

3. Introduction: Expand the discussion on how this study differs from or builds upon previous research in other countries. This will help clarify the novelty of the study and its contribution to the global literature. Provide a more detailed explanation of how each hypothesis is derived from the theoretical framework or past studies to strengthen the foundation of the research.

   Thank you for your suggestion. In response, we have expanded the Introduction section to better highlight how this study differs from or builds upon previous research conducted in other countries. We have provided a more detailed discussion of the novelty of this study and its contribution to the global literature, particularly in the context of urban youth in India.

   This study uniquely addresses the impact of nature connectedness on the mental health and well-being of urban youth in India, a context that has been underexplored in the existing literature, especially when compared to studies conducted in Western countries. While there is considerable research on the benefits of nature connectedness in countries such as the United States, Canada, and the United Kingdom, studies focusing on the urban youth demographic in India are scarce. Our study fills this gap by examining the relationship between nature connectedness and well-being within a rapidly urbanizing, diverse, and culturally distinct context.

   We have also elaborated on how each hypothesis is derived from the theoretical framework and past studies. For example, the hypothesis that nature connectedness positively influences well-being is rooted in the Attention Restoration Theory (ART) and biophilia hypothesis, which have been well-established in research across various cultures. Building on these frameworks, we hypothesized that urban youth in India, much like in other countries, would benefit from nature exposure.

   Additionally, the hypothesis linking access to nature and mental health is supported by previous studies from both Western and Asian contexts that highlight the role of green spaces in reducing stress and promoting psychological well-being. By grounding these hypotheses in established theories and research, we strengthen the foundation of our study and demonstrate how our findings could extend and contribute to global conversations about the role of nature in enhancing mental health, particularly for urban youth.

   These revisions help clarify the unique contribution of this study to the global literature on nature connectedness and youth well-being, while also providing a more robust theoretical underpinning for each hypothesis.

4. Materials and Methods: Acknowledge the sampling bias more explicitly in this section and discuss potential strategies for mitigating this issue in future studies, such as stratified sampling or oversampling underrepresented cities. Include a discussion on the cultural relevance of the WHO-5 and NIS scales in the Indian setting and any modifications made to ensure their validity and reliability. Suggest alternative or complementary data collection methods, such as observational data or longitudinal tracking, to strengthen the study's validity and reduce reliance on self-report. Provide a more detailed justification for the inclusion of specific covariates and discuss any additional variables that might have been considered.

   Thank you for your suggestions. We address these points in the Limitations and recommendations for future research section.

   We have explicitly acknowledged the potential for sampling bias due to the uneven distribution of participants across cities. To mitigate this issue in future studies, we suggest employing stratified sampling or oversampling underrepresented cities to ensure a more balanced and representative sample.

   We adopted the international versions of the WHO-5 and NIS scales without modification. These scales are widely used and validated in various international settings, and we ensured their applicability in the Indian context based on their proven reliability and validity across diverse populations.

   To strengthen the study's validity and reduce reliance on self-report data, we have suggested incorporating alternative or complementary data collection methods, such as observational data

or longitudinal research design. These approaches could provide deeper insights into participants' behaviors and experiences over time.

Finally, we have provided a more detailed justification for the inclusion of specific covariates in the study. We also discuss additional variables that could have been considered in future research to further enrich the analysis and account for more potential confounding factors.

These points are elaborated in the limitations and recommendations for future research section, where we highlight strategies for improving future research in this area.

5. Results: Include more context or commentary on the significance of the descriptive statistics, especially how they relate to the urban youth population in India. Expand the discussion on the counterintuitive results, such as the negative association with blue spaces, by exploring potential cultural or environmental factors specific to the Indian context. Discuss the limitations of the city-specific analysis due to uneven sample sizes and consider recommending further research to validate these findings with more balanced samples.

   Thank you for your suggestions. As mentioned in our earlier response, we have expanded the Discussion section to include more context on the significance of the descriptive statistics, particularly in relation to the urban youth population in India. We have also explored potential cultural and environmental factors contributing to the counterintuitive negative association with blue spaces, considering factors such as pollution, accessibility, and safety concerns specific to the Indian context.

   Additionally, we have acknowledged the limitations of the city-specific analysis due to uneven sample sizes and recommended employing more balanced sampling strategies, such as stratified sampling, in future research to ensure more representative results. These points have been addressed in both the Discussion and Limitations and Recommendations for Future Research sections.

6. Discussion: Provide a more in-depth discussion of the limitations, particularly the sampling bias and the reliance on cross-sectional data, and suggest ways these could be addressed in future research. Include a more comprehensive comparison with international studies, highlighting both the similarities and differences, and discuss the implications for cross-cultural research on nature connectedness. Provide more concrete examples of how urban planners and policymakers can use the findings to design interventions that promote nature connectedness among urban youth.

   Thank you for your suggestions. In response, we have rewritten the Discussion section to provide a more in-depth analysis of the limitations, particularly focusing on the sampling bias and the reliance on cross-sectional data. We have discussed how these limitations could impact the validity of the findings and suggested strategies to address them in future research, such as employing longitudinal designs and more balanced sampling methods like stratified sampling to reduce bias.

   Additionally, we have included a more detailed discussion on the practical implications of the study for urban planners and policymakers. We provide concrete examples of how the findings can be used to design interventions that promote nature connectedness among urban youth, such as creating more accessible green spaces, integrating nature into urban planning, and encouraging outdoor activities.

   These updates have been incorporated into the Discussion section, with appropriate sub-sections to clearly address each of the points raised.

7. Conclusion: Streamline the conclusion to emphasize the key contributions and their implications for both the scientific community and policymakers. Propose specific future research directions that address the study's limitations, such as exploring seasonal variations, using a longitudinal design, or incorporating qualitative methods.

   Thank you for your suggestions. We have streamlined the conclusion to emphasize the key contributions and their implications for both the scientific community and policymakers, while also proposing specific future research directions to address the study's limitations.

Reviewer 2

The manuscript demonstrates technically sound scientific research, with data to support the conclusions presented. The experiments were conducted rigorously, using appropriate controls, replication and adequate sample sizes, allowing conclusions to be drawn appropriately based on the data presented.

The conclusions are carefully drawn from the data presented and corroborate the initial hypotheses. The study shows that residential proximity to green spaces, frequent recreational visits to these spaces and a strong connection with nature are significantly associated with better mental well-being, while spending more time indoors has a negative impact on mental health. some pointers to improve the manuscript can be found in the attached documents (Copied here for reference).

1. Address the issue of sampling bias in the text, including a more in-depth discussion of the implications of this bias on the results. Sample imbalance can affect the result and it would be important to detail the implications in the study.

   Thank you for raising this important point. We acknowledge that the number of responses across the 25 cities in our study is uneven, which could introduce sampling bias. This uneven distribution is primarily due to differences in population sizes, variations in survey outreach effectiveness, and logistical challenges faced in certain cities. For example, larger cities like Delhi and Mumbai naturally yielded more responses due to their higher populations and better survey reach, whereas smaller cities like Rajkot received fewer responses due to limited outreach and other factors.

   We have added an explanation of this issue in the Limitations section, where we discuss the potential impact of this sampling imbalance on the representativeness of the data. Additionally, we have outlined strategies to address this in future research, such as employing stratified sampling or oversampling underrepresented cities to ensure a more balanced and accurate representation of the urban youth population across India.

2. Can there be seasonal variations in the collection of results? Does the data collected allow this to be visualized? If so, can this be explored in the paper?

   Thank you for highlighting the potential impact of seasonal variations on outdoor activities and nature connectedness. We acknowledge that this is a limitation of our study, as data collection was conducted during a specific period and did not account for seasonal differences. Due to logistical constraints, time and funding limitations, it was not feasible to capture responses across multiple seasons. While this may introduce some bias, it is important to note that the focus of this study was on broader trends rather than season-specific behaviors. We have addressed this limitation in the Limitations section.

   In future research, we plan to incorporate longitudinal data collection across different seasons to better capture seasonal variations and their potential impact on nature connectedness and well-being. This would allow us to explore these seasonal differences more thoroughly and provide more nuanced insights into how changes in seasons influence urban youth's relationship with nature.

3. Although the study acknowledges some limitations, such as the crosssectional nature of the research and the possibility of bias in the data, the discussion of these issues is relatively brief, it would be important to include a more critical and in-depth analysis of the study's limitations and their potential implications for the conclusions. It is important to present the limitations transparently and discuss how they may influence the interpretation of the results.

   Thank you for your suggestions. In response, we have thoroughly revised the Discussion section to provide a more critical and in-depth analysis of the study's limitations and their potential implications for the conclusions. We have added a dedicated Limitations and recommendations for future research sub-section, where we transparently discuss key limitations such as the cross-sectional nature of the research, uneven sample across cities, and other potential study limitations and how to address them in future research. We have also elaborated on how these limitations could influence the interpretation of the results and the generalizability of our findings. This revision aims to provide a more balanced perspective on the study's strengths and constraints.

4. The study focuses mainly on the statistical analysis of the relationship between connection with nature and mental well-being, it would be important to explore the practical implications of

the study for urban planning, public mental health policies and community interventions. It is important to discuss how the results can be used to promote the mental health and well-being of the urban population through the creation of and access to green and blue spaces. Presenting the practical implications of the study makes the research more relevant and useful.

Thank you for your suggestions. We agree that translating our findings into actionable strategies is essential to enhance the relevance and utility of this research. We have added a sub-section Practical Implication to our Discussion section. Our study, focusing on urban youth, underscores the positive relationship between connection with nature and well-being, which has significant implications for urban planning, public mental health policies, and community interventions. Urban youth, particularly in fast-paced, high-stress environments, are increasingly experiencing mental health challenges due to factors like pollution, limited access to nature, and social isolation. In this context, enhancing the availability and accessibility of green and blue spaces becomes crucial. Urban planners should prioritize creating parks, gardens, and water bodies within city scapes, ensuring that these spaces are not only available but also equitably distributed across neighbourhoods, providing all urban youth with the opportunity to connect with nature. Designing restorative urban environments—such as tree-lined streets, green rooftops, and nature corridors—can further help mitigate urban stressors and promote mental well-being for young people navigating the complexities of city life.

In terms of public mental health policies, our study suggests that integrating nature-based interventions can play a vital role in promoting mental health among urban youth. Public health campaigns could include nature walks, forest therapy programs, or outdoor community activities tailored to engage young people and reduce stress. Policies should advocate for the preservation and expansion of green spaces within urban areas, viewing nature not just as a luxury, but as a crucial element for the psychological well-being of youth. On the community level, interventions such as urban gardening, eco-volunteering, and nature-based recreational programs can foster a stronger sense of community and connection to the environment. These initiatives can serve as both mental health interventions and tools for youth empowerment, giving them the opportunity to engage meaningfully with their surroundings. Additionally, educational programs in schools and community centres can raise awareness about the mental health benefits of nature, encouraging young people to regularly engage with green spaces. These practical applications of our research offer a clear path forward to improve the mental health and well-being of urban youth, creating healthier, more resilient communities in urban environments.

Reviewer 3

1. Although the study is methodologically well-structured and employs multiple regression and logistic regression models, there are some shortcomings in the hypothesis section. The research could strengthen its hypotheses by providing more detailed theoretical foundations. Furthermore, the discussion on the negative correlation between blue spaces (e.g., bodies of water) and mental health is rather superficial. It is recommended to delve deeper into the possible reasons for this result in the discussion section, such as the impact of urban geographical location, urban planning, or other socio-cultural factors, and to cite more relevant literature to support this discussion. This would make the conclusions more reasonable and convincing.

   Thank you for your insightful feedback. We appreciate your suggestion to strengthen the theoretical foundations of our hypotheses. In response, we have revised the Hypotheses section to provide a more detailed explanation of the theoretical frameworks and past studies that informed our research questions. Regarding the negative correlation between blue spaces and mental health, we agree that this result warrants a more thorough discussion. In the revised Discussion section, we have expanded on possible reasons for this unexpected finding.

2. The study is based on self-reported survey data, which introduces potential biases and challenges in validation. As the authors have mentioned, the sample distribution across different cities is uneven, and the sample size in some cities is too small, which may affect the generalizability of the research results. Uneven sample distribution among different cities may impact the generalizability

of the results, especially in cities with extremely small sample sizes. It is suggested to explain the sample selection strategy in detail in the methods section and to further discuss in the limitations how this issue affects the research results. Future research could consider allocating samples more evenly across different cities.

Thank you for highlighting the potential impact of uneven sample distribution and the challenges related to self-reported data. We acknowledge that the sample distribution across the 25 cities in our study is uneven, which could affect the generalizability of the results, especially in cities with very small sample sizes. The uneven distribution was primarily due to differences in population sizes, the effectiveness of survey outreach and logistical constraints in certain cities, and also overall budget and time constrains for the study.

To mitigate potential biases, we designed the survey to ensure participant anonymity, which reduces the likelihood of socially desirable responses. Furthermore, we used well-validated and widely accepted scales to measure nature connectedness and well-being, aiming to minimize bias and enhance reliability.

We have now provided a more detailed explanation of the sample selection strategy in the Materials and Methods section. Additionally, we have discussed in the Limitations section how the uneven sample distribution may impact the generalizability of the findings, and we suggest that future research consider more evenly distributed samples across cities to address this issue.

3. Since this manuscript is based on self-reported data, there may be biases. Incorporating more indigenous studies on the mental health of urban adolescents in India and their connection with nature would further enhance the background and relevance of the research. Although the citation of global studies is sufficient, the article could improve its quality by integrating more regional data and research. For example, providing more explanations about the data collection process, questionnaire design, and how biases were controlled, and including key questions from the questionnaire in the appendix, would further enhance the transparency of the data and the scientific rigor of the research.

   We appreciate the reviewer's insightful comments. Although we recognize the importance of incorporating indigenous studies on urban youth and their connection with nature in India, the limited availability of such studies was a constraint. However, we have acknowledged this gap in the manuscript to emphasize the need for more regional research in this area. Furthermore, we have elaborated on the data collection process, questionnaire design, and measures taken to minimize biases as follows:

   Data collection process:

   Target population: Urban youth aged 18-25 years of age from 25 major Indian cities. Sampling method: Convenience sampling through academic institutions, social media platforms, and local community networks. Mode of data collection: Online survey for wider reach and accessibility. Ethical considerations: Informed consent obtained; confidentiality and anonymity ensured.

   Questionnaire design:

   Validated scales: Psychometrically validated tools, including the WHO-5 well-being index and inclusion of nature in self scale to measure nature connectedness. Customization: Minimal language adaptation for cultural relevance. Pretesting: Pilot test conducted to refine questions and resolve ambiguities.

   Measures to minimize biases:

   Anonymity: Survey responses were anonymous to reduce social desirability bias. Neutral wording: Questions were phrased neutrally to avoid leading responses. Randomized item order: Items were randomized to minimize response patterns. Standardized instructions: Clear instructions provided for uniform understanding.

Reviewer 4

1. Please define the scope of 'natural connection', what does it include? This will help the understanding of scholars not in the field.

Thank you for this suggestion. "Natural connection" refers to an individual's sense of belonging and affinity for the natural world, including awareness, emotional bonds and actions that foster a relationship with nature. It includes mindfulness in nature, sustainable behaviors, and recognition of interdependence with ecosystems, making it integral to well-being and environmental psychology.

2. please provide justification for the choice of this city.

   The choice of the 25 cities included in this study was based on their urban population size, which allowed us to capture a diverse and representative sample of urban youth across India. These cities are among the most populated in India and were selected to reflect the urban youth population's variation in socio-economic, cultural, and environmental contexts. By focusing on large cities, we aimed to ensure that the findings could offer insights relevant to the broader urban youth population in India.

   Furthermore, the selection of these cities was driven by practical considerations, including the feasibility of reaching a sufficient sample size, access to survey participants, logistical and budget considerations. While the uneven sample distribution across cities is acknowledged as a limitation, this selection strategy ensured the inclusion of key urban areas with varying demographic and geographic characteristics, enhancing the diversity and relevance of the study's findings.

3. is the study of India useful for urban studies in other countries?

   The study of urban youth in India offers valuable insights that can contribute to urban studies in other countries, especially those experiencing similar challenges related to urbanization, youth well-being, and access to nature. While the specific findings may be influenced by India's unique urban context, the research provides a comparative perspective that can inform global discussions on urban health and environmental factors. The relationship between urbanization, nature connectedness, and well-being in Indian cities can offer lessons that are applicable to rapidly urbanizing regions worldwide.

   While India's cultural context differs from other countries, the pressures faced by urban youth—such as those related to education, employment, and social connectivity—are universal. The study highlights trends in youth well-being that may be present in other urban environments, encouraging cross-cultural comparisons. These findings can prompt further exploration into how urban environments impact mental health and social connectedness in youth across various countries.

   Moreover, the research provides policy implications that could resonate globally, particularly regarding urban planning strategies to promote well-being. Integrating nature into urban spaces, such as the inclusion of green and blue spaces, is a concept that is relevant to cities around the world. While the findings may not be directly generalizable to all countries, the study's methodology and approach can serve as a model for future research in urban environments globally, helping policymakers create more supportive spaces for urban youth.

Additional comments by the editor

1. Elaborate on the sample selection strategy in the Methods section and explore the impact of this distribution on the findings in the Discussion section.

   We have revised the Methods section to include a detailed explanation of the sample selection strategy. Specifically, we have outlined the criteria for selecting the 25 cities, emphasizing their high population and urbanization levels. The impact of uneven sample distribution on the findings has been addressed in the Discussion section, highlighting its implications for generalizability and potential biases in city-specific analyses.

2. Strengthen the theoretical support for the hypotheses, particularly by discussing the basis for the hypotheses in the context of the existing literature.

   The Introduction section has been expanded to provide stronger theoretical foundations for the hypotheses. We have drawn from global and regional studies to illustrate the established relationships between nature connectedness, mental health, and well-being, specifically emphasizing cultural and geographical factors relevant to the Indian context.

This includes studies that demonstrate the psychological benefits of green and blue spaces, as well as literature on youth mental health in urban settings.

3. Explain the data collection process and bias control measures in more detail in the Methods section.
   The Methods section now includes a comprehensive account of the data collection process. This includes details on the sampling strategy (convenience sampling), outreach channels (academic institutions, social media, and community networks), and ethical safeguards (anonymity and informed consent). Bias control measures, such as the use of validated scales, neutral wording of survey items, randomized item order, and pretesting, have also been elaborated upon.
4. Insufficient explanation of the negative correlation between blue space and mental health.
   We acknowledge this counterintuitive finding and have expanded the Discussion section to explore possible reasons for the negative correlation. This includes the role of pollution, poor maintenance of blue spaces, and limited cultural significance of water bodies in urban Indian contexts. Comparative insights from international studies have also been added to provide a nuanced interpretation.
5. Discuss the limitations of cross-sectional study design and uneven sample distribution.
   A dedicated subsection on limitations has been included in the Discussion section. We have addressed the challenges posed by the cross-sectional design, which limits causal inferences, and the uneven sample distribution, which affects generalizability. We have also proposed potential solutions, such as longitudinal studies, stratified sampling, and geographically balanced data collection in future research.
6. Add a comparative analysis with international studies to clarify the unique contribution of this study in a global context.
   The Discussion section has been expanded to include a comparative analysis with international studies from Canada, Europe, the United States, and Asia. This highlights both common trends (e.g., positive impact of green spaces on well-being) and unique findings (e.g., the role of cultural perceptions of blue spaces in India), showcasing the global relevance and regional specificity of our study.
7. Define the scope of “natural connections” and explain why these cities were selected and their value for other urban studies.
   We have defined “natural connections” in the context of this study as the psychological, emotional, and behavioral relationships individuals have with natural environments. The rationale for selecting the 25 most populated cities in India is discussed in the Methods section, emphasizing their representativeness of urban youth experiences in diverse geographical and cultural settings. We have also elaborated on the implications of these findings for urban studies in other countries with rapidly urbanizing populations.
8. Add specific recommendations for urban planning and policy applications and expand the literature review to include more indigenous Indian studies and regional data.
   The Conclusion section now includes targeted recommendations for urban planning, such as increasing the availability of accessible green and blue spaces, integrating nature-based interventions (e.g., community gardening, outdoor mindfulness activities), and incorporating natural elements into urban design. The literature review has been updated to include more indigenous studies and region-specific data, strengthening the contextual relevance of the findings.
