# Supplementary material for: Exploring the impact of nature connectedness on well-being and mental distress among urban youth: Evidence from 25 most populated cities in India
Source: PLoS One. 2025 May 30;20(5):e0323712. doi: 10.1371/journal.pone.0323712 (PMC12124530; doi:10.1371/journal.pone.0323712)
Supplement: Attachment — Submitted filename: Responses to reviewers_revision 2.pdf [file pone.0323712.s003.pdf]

We want to thank the reviewers for their valuable suggestions in improving the article. We have added our responses to each comment below. We have also made relevant changes in the manuscript.

Journal requirements

Please review your reference list to ensure that it is complete and correct. If you have cited papers that have been retracted, please include the rationale for doing so in the manuscript text, or remove these references and replace them with relevant current references. Any changes to the reference list should be mentioned in the rebuttal letter that accompanies your revised manuscript. If you need to cite a retracted article, indicate the article's retracted status in the References list and also include a citation and full reference for the retraction notice.

Thank you for your suggestion. We have carefully reviewed the reference list to ensure its accuracy and completeness. In doing so, we identified and removed any retracted references, replacing them with relevant and current sources where applicable. These changes have been reflected in the revised manuscript.

Reviewer 4

1. Please improve the quality of all Figures. the text in Figure 8 is too small, making it difficult to read the content.

   Thank you for your feedback. We have improved the quality of all figures. We have removed city names from Figure 7 and have replaced them with colors. Each color represents a city now.

2. "age", "gender", "education", and "profession" in the bar chart can be used as subfigures to form a completed chart.

   Thank you for your suggestion. We have reorganized the bar charts by combining the covariates as subfigures of a larger main figure. This is as given in Figure 2.

3. Please check the spelling of the second author in References 59.

   Thank you for your observation. We have checked and corrected the spelling of the second author.

4. It is recommended that an academic contribution map be drawn in "Discussions" to illustrate the contribution of the research to the field as a whole.

   Thank you for your suggestion. We have clarified our research contributions in the Discussion section and structured them for better readability.

5. Also, in Discussions, explain how the research will be replicated and disseminated. Include the study in the local area, as well as the impact on the global scale.

   Thank you for your suggestion. We have expanded the Discussion section to explain how the study can be replicated and disseminated, considering both local applications and its broader global impact.

6. Integrate theoretical frameworks, such as the Biophilia Hypothesis or Attention Restoration Theory, in the Introduction section as theoretical support for subsequent research.

   Thank you for your insightful suggestion. We have integrated theoretical frameworks, including the Biophilia Hypothesis, Stress Reduction Theory, and Attention Restoration Theory, in the Introduction section to strengthen the theoretical foundation of our research.

7. Refine the logical relationship between "natural connectivity" and "mental health" and explain its possible mediating or moderating variables.

   Thank you for your suggestion. While our study explores the relationship between nature connectivity and mental health, we acknowledge the need to consider potential mediating or moderating variables. Since these variables were not tested in the current study, we have included this aspect under recommendations for future research.

8. How is the variable “indoor time” defined and measured? The authors may add to the list.

   Thank you for your suggestion. We have clarified the definition and measurement of ”indoor time” within the covariates section in the manuscript.

9. Compare and contrast the results of domestic and international studies, analyze the possible reasons behind the differences, and highlight the academic contributions of this study.

   Thank you for your suggestion. As there are limited domestic studies on this topic, we have primarily compared our findings with international research while acknowledging the gap in local literature. We have also discussed possible reasons for any differences observed and highlighted the academic contributions of our study in addressing this research gap.
